# Chronic radiation proctitis refractory to steroid enema was successfully treated by metformin and sodium butyrate: a case report

**DOI:** 10.1186/s13256-024-04551-x

**Published:** 2024-05-10

**Authors:** Mau-Shin Chi, Ping-Hsun Hsieh, Shu-Han Huang, Ho-Chi Hsu, Kwan-Hwa Chi

**Affiliations:** 1 Department of Radiation Therapy and Oncology, Shin Kong Wu Ho-Su Memorial Hospital, Taipei, Taiwan; 2 Institute of Veterinary Clinical Science, School of Veterinary Medicine, National Taiwan University, Taipei, Taiwan; 3 Division of Gastroenterology, Department of Internal Medicine, Shin Kong Wu Ho-Su Memorial Hospital, Taipei, Taiwan; 4 Department of Pathology and Laboratory Medicine, Shin Kong Wu Ho-Su Memorial Hospital, Taipei, Taiwan; 5 Department of General Surgery, Shin Kong Wu Ho-Su Memorial Hospital, Taipei, Taiwan

**Keywords:** Case report, Chronic radiation proctitis, Radiotherapy, Metformin and butyrate enema, Metformin, Sodium butyrate

## Abstract

**Background:**

Radiation proctitis (RP) is a significant complication of pelvic radiation. Effective treatments for chronic RP are currently lacking. We report a case where chronic RP was successfully managed by metformin and butyrate (M-B) enema and suppository therapy.

**Case presentation:**

A 70-year-old Asian male was diagnosed with prostate cancer of bilateral lobes, underwent definitive radiotherapy to the prostate of 76 Gy in 38 fractions and six months of androgen deprivation therapy. Despite a stable PSA nadir of 0.2 ng/mL for 10 months post-radiotherapy, he developed intermittent rectal bleeding, and was diagnosed as chronic RP. Symptoms persisted despite two months of oral mesalamine, mesalamine enema and hydrocortisone enema treatment. Transition to daily 2% metformin and butyrate (M-B) enema for one week led to significant improvement, followed by maintenance therapy with daily 2.0% M-B suppository for three weeks, resulting in continued reduction of rectal bleeding. Endoscopic examination and biopsy demonstrated a good therapeutic effect.

**Conclusions:**

M-B enema and suppository may be an effective treatment for chronic RP.

## Background

Chronic rectal toxicity is a significant concern after primary radiotherapy for the prostate, cervix or rectal cancer [1, 2]. Incidence rises with higher radiation doses and irradiated volumes to the rectum [3], with late grade 2 rectal toxicity reported between 5 to 21% [3–5]. Zimmermann and Feldmann noted the lack of proven effective preventive measures for late radiation proctitis (RP) [6]. The effectiveness of commonly used treatments such as corticosteroids [7], sucralfate [8], 5-aminosalicylic acid[9, 10] remains inconclusive. Butyrate, a short-chain fatty acid primarily produced by anaerobic bacteria from dietary fiber in the colon, serves as the primary energy source for colonocytes [11]. Although butyrate enemas have shown success in acute RP [12, 13], their efficacy in chronic cases is less certain [14, 15]. The pathogenesis of chronic RP primarily involves vascular ectasia [16]. Inspired by metformin's success in treating diabetic retinal angiopathy, we propose the utilization of combined metformin and butyrate (M-B) enema for chronic RP [17, 18]. Here, we present a case of chronic RP resistant to conventional therapies, which subsequently responded to M-B enema therapy.

## Case presentation

A 70-year-old Asian male presented with intermittent rectal bleeding lasting for 2 months. He had type 2 diabetes and hypertension on regular medicine with metformin 500 mg twice daily and amlodipine / valsartan 5/160 mg daily. Additionally, he had a history of benign prostatic hypertrophy with rising PSA levels up to 11.9 ng/mL. He underwent Diode Laser enucleation to address low urinary obstruction symptoms, and a trans-rectal needle biopsy reported prostate adenocarcinoma involving bilateral lobes. The Gleason score was 4 + 3 on the left side and 3 + 4 on the right side, with a clinical stage of cT2cN0. He underwent a six-month course of androgen deprivation therapy with Goserelin injections, followed by primary radiotherapy using TomoTherapy (Accuray, Sunnyvale, CA, USA), delivering a total dose of 76 Gy in 38 fractions after one month of neoadjuvant hormone therapy. His PSA levels reached a nadir of 0.2 ng/mL and remained stable for 10 months post-radiotherapy until the onset of intermittent rectal bleeding. Colonoscopy revealed mucosal changes indicative of RP. Initial conservative management with oral mesalamine, mesalamine enema and hydrocortisone enema was administered for two months, providing temporary relief, but rectal urgency, and intermittent small amounts of rectal bleeding, persisted.

**Fig. 1:**
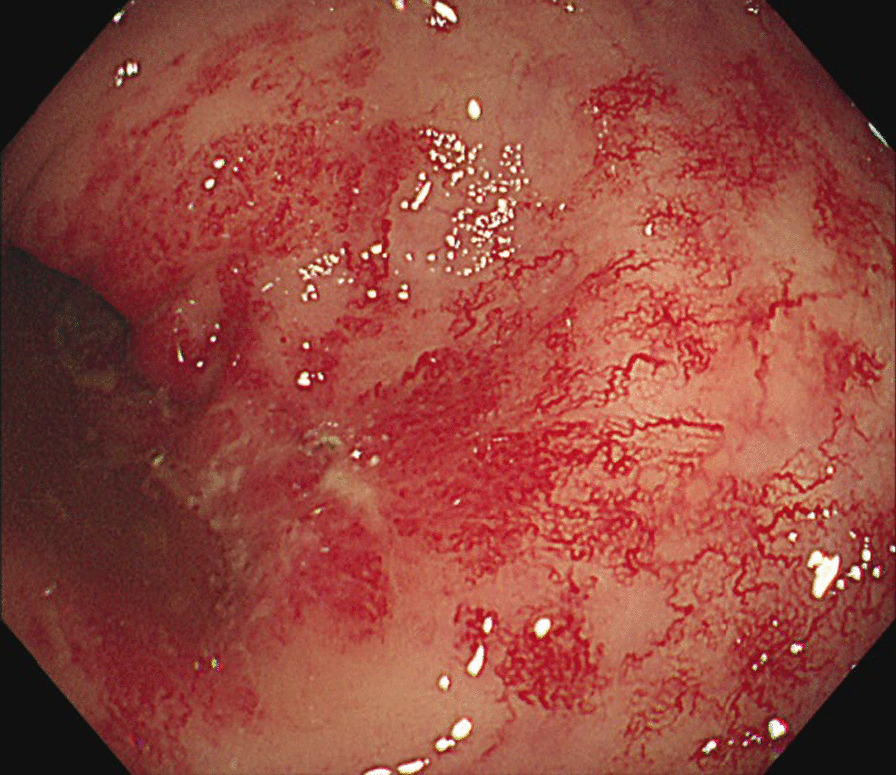
Diagnosis of chronic radiation proctitis. Diffuse angioectasia with congested and edematous rectal mucosa, characteristic of radiation proctitis. There was small ulceration and active bleeding noted

**Fig. 2:**
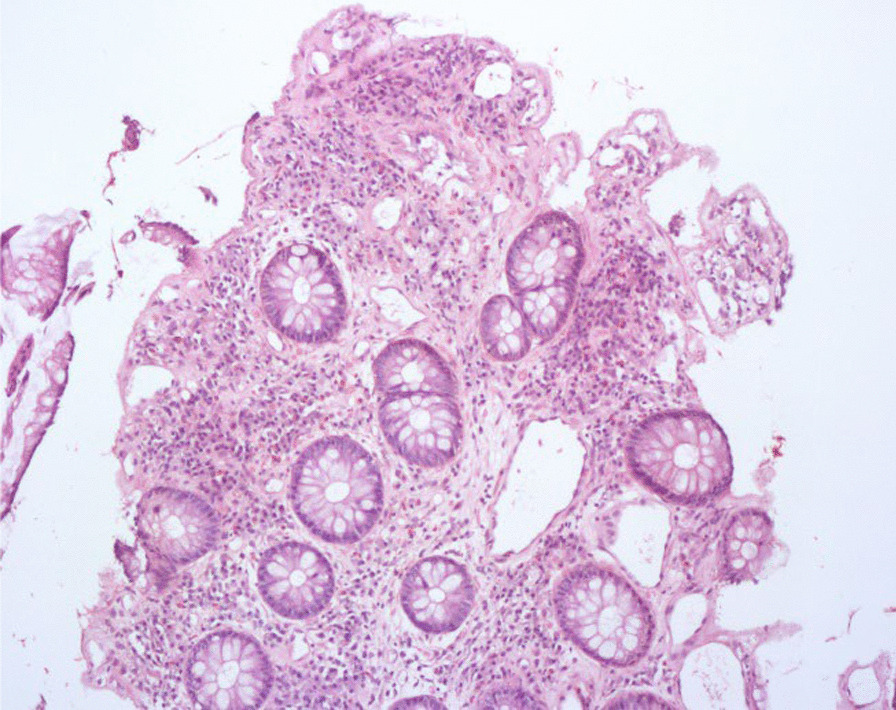
Histopathological changes before treatment. The mucosal surface is erosive. Dense inflammatory cells infiltrate in the mucosa are noted. (hematoxylin–eosin (HE) 25X)

His vital signs were stable, and the physical examination was unremarkable, aside from the presence of fresh blood during digital examination. Laboratory findings indicated elevated fasting plasma glucose and HbA1c levels consistent with diabetes, while hemoglobin, white blood cell count, platelets, and liver function tests were all within normal ranges. PSA levels remained at 0.2 ng/mL. Colonoscopy revealed diffuse angioectasia, edematous rectal mucosa, and small ulceration with blood over the recto-sigmoid junction, consistent with RP (Fig. 1). Biopsy showed mucosal surface erosion with dense inflammatory cell infiltrate (Fig. 2), confirming a diagnosis of grade 2 chronic RP.

**Fig. 3:**
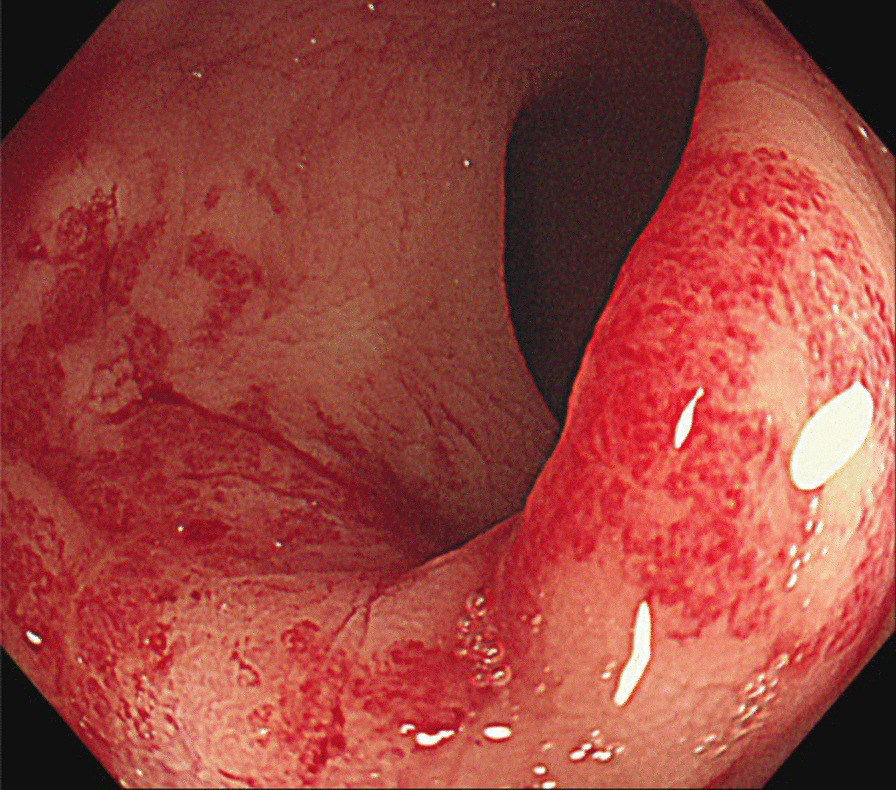
Sigmoidoscope follow-up one month after treatment. Follow-up sigmoidoscopy showing improvement with lesser extent of telangiectasia and erythema. The ulceration was healed

**Fig. 4:**
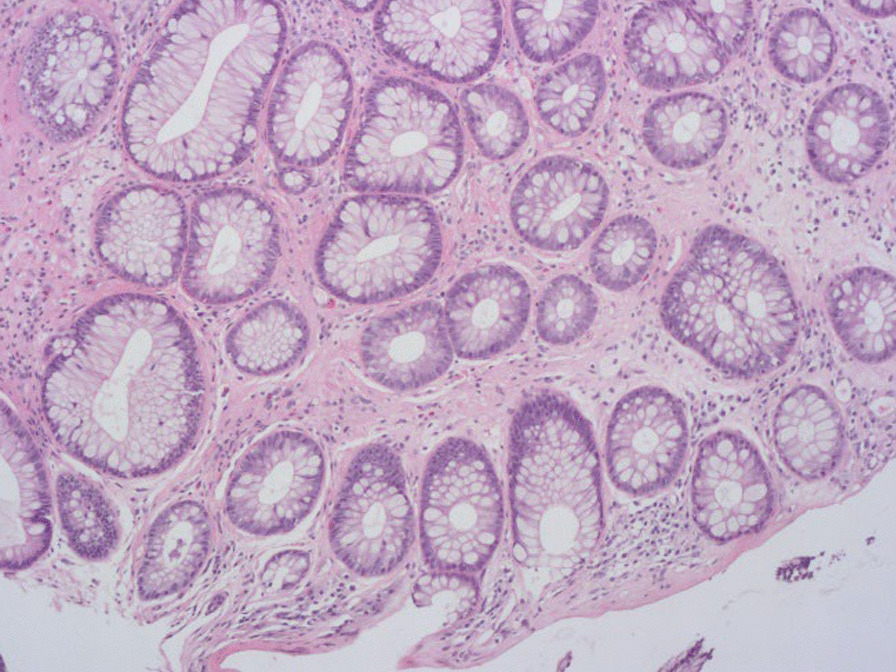
Histopathological changes after treatment. The number of inflammatory cells is decreased in the mucosa. (HE 25X)

He received treatment with a novel enema formulation, applying 2% M-B (metformin and sodium butyrate) daily in a total of 60 ml per enema treatment. The M-B suppository, comprising 2.0% metformin and sodium butyrate, was coated with cocoa butter and molded into 2.5 Gm suppositories. Remarkably, his bloody stools improved within just one week of starting this treatment. He continued maintenance therapy for an additional three weeks, resulting in significant clinical improvement, with cessation of rectal bleeding and only mild remaining proctitis symptoms (grade 1). One month after initiating M-B enema therapy, he underwent an endoscopic examination and biopsy. While no ulceration was observed, the density of telangiectasia remained unchanged; however, there was a reduced extent of rectal mucosal congestion and hypervascularity (Fig. 3). The histopathological analysis revealed an improvement in inflammatory cell infiltration (Fig. 4).

## Discussion

We present the first documented case in medical literature of effectively managing chronic RP with M-B enema and suppository therapy. The rapid alleviation of rectal bleeding within a week of initiating M-B enema highlights its effectiveness in managing this challenging condition through a noninvasive approach. Management of chronic RP has long been challenging, due to lack of solid, randomized clinical trials. Many commonly recommended medical interventions for chronic RP lack robust evidence from large-scale studies. In cases resistant to standard treatments like steroids, mesalamine, sulfasalazine, sucralfate, or butyrate enemas, the American Society of Colon and Rectal Surgeons guideline suggests considering endoscopic argon plasma coagulation [19].

From a pathological perspective, chronic RP is characterized by radiation-induced vascular abnormalities intertwined with inflammation, termed radiation-associated vascular ectasia. Many medications used in chronic RP management possess anti-inflammatory properties. Metformin, known for its role in wound healing and angiogenesis, activates AMP-protein kinase and facilitates epithelial barrier recovery [20, 21]. Additionally, butyrate exerts anti-inflammatory effects on the intestinal mucosa, promoting tissue repair and regeneration through epithelial cell proliferation stimulation [22]. The M-B synergism may contribute to restoring epithelial homeostasis and normalizing vascular ectasia in chronic RP [23].

In the context of inflammatory responses, both classical M1 macrophages and alternatively activated M2 macrophages play pivotal roles [24]. While M1 macrophages drive pro-inflammatory responses, M2 macrophages contribute to tissue repair and anti-inflammatory functions [25]. Metformin and butyrate exhibit anti-inflammatory effects on M1 macrophages by reducing proinflammatory mediators, such as NO or IL-6, thereby facilitating M2 polarization [26, 27]. One hypothesis suggests that M-B combination might directly enhance M2 macrophage polarization, while another suggests it may indirectly prevent M2 macrophage senescence. Radiation has been implicated in accelerating M2 macrophage senescence, leading to the secretion of inflammatory cytokines and perpetuating tissue inflammation [28]. However, the precise mechanism by which M-B prevents M2 macrophage senescence requires further investigation.

A proactive prevention strategy holds theoretical promise over a reactive approach. There is a recognized correlation between acute and late rectal toxicities, suggesting that preventing acute RP may mitigate the onset of chronic RP [29]. Given that acute RP typically self-resolves, prevention during and after radiotherapy may avert the development of chronic RP. Use of M-B suppositories for preventive purposes presents a potentially convenient alternative to enemas. Further study is warranted to explore the M-B suppositories in greater depth.

## Conclusion

Here we report the first case to alleviate chronic RP with M-B suppository therapy. The synergistic effect of M-B may offer promise in normalizing radiation-associated vascular ectasia and preventing M2 macrophage senescence. We believe these findings may warrant further investigation of an official clinical trial in a multi-institutional setting.

## Data Availability

All data generated or analysed during this study are included in this published article.

## Abbreviations

RP: Radiation proctitis
M-B: Metformin and butyrate
